# Paradoxical Emboli Secondary to Hepatic Pathology: Common or Coincidental?

**DOI:** 10.1155/2009/184192

**Published:** 2009-11-22

**Authors:** Patrik Gabikian, Melanie Walker, Abhineet M. Chowdhary, Arthur M. Lam, Gavin W. Britz

**Affiliations:** ^1^Department of Neurological Surgery, University of Washington School of Medicine, Seattle, WA 98104-2420, USA; ^2^Department of Neurology, University of Washington School of Medicine, Seattle, WA 98104-2420, USA; ^3^Department of Anesthesiology, University of Washington School of Medicine, Seattle, WA 98104-2420, USA; ^4^Division of Neurosurgery, Duke University School of Medicine, Durham, NC 27710, USA

## Abstract

Paradoxical cerebral emboli from cardiac and pulmonary sources are well described in the peer-reviewed literature. We outline a case with a hepatic etiology and describe diagnostic and management options. Though this paper represents the first documentation of such, we believe that transpulmonary shunting with concurrent paradoxical cerebral microemboli is more prevalent than recognized. We introduce this case report to compel practitioners to consider paradoxical emboli in selected cirrhotic patients since it can often be difficult to elicit subtle neurologic changes on clinical examination of patients with end stage liver disease.

## 1. Introduction

While cardiogenic emboli are well described, paradoxical emboli resulting from hepatic disease have not been discussed in the neurologic literature. We describe a complicated patient case, where brain emboli were incidentally discovered, and briefly discuss the underlying pathology responsible for this extracardiac phenomenon.

## 2. Case Presentation

A 60-year-old woman presented to our medical center for evaluation of acute onset headache and confusion. Her past medical history was remarkable for alcoholic cirrhosis and hepatitis C. Physical examination revealed the presence of mild ascites and hypoxemia (91%) on room air with no response to supplemental oxygen. Noncontract head CT was without focal abnormality, but cerebrospinal spinal fluid analysis showed xanthrochromia suggestive of subarachnoid hemorrhage. Catheter angiography of the cerebral vessels confirmed the presence of a right middle cerebral artery (MCA) aneurysm, and the patient underwent endovascular coiling without complication. Postprocedure day number two, the patient decompensated and developed respiratory and congestive heart failure. A right internal jugular catheter was placed for venous access and hemodynamic monitoring. Daily surveillance transcranial Doppler (TCD) and noncontrast head CT showed no abnormality until postprocedure day number four when embolic signatures were detected in the basilar and bilateral MCAs. Given the multiple vascular territories involved in the ultrasound findings, a search for the source of emboli was initiated. The patient was unable to undergo transesophageal echocardiogram due to the presence of varices, but transthoracic echocardiogram with agitated saline contrast was performed while mechanically ventilated and repeated once she was extubated. No patent foramen ovale was identified, but a large amount of echogenic contrast was detected in the left atrium after 3-4 heartbeats. The delayed appearance of saline contrast in the left atrium and its origination from the right pulmonary vein were anatomically consistent with shunting through the pulmonary circuit, and similarly confirmed by TCD. The patient was started on daily antiplatelet therapy with 325 mg aspirin and the embolic signatures on TCD ceased within three days. She soon recovered from the events surrounding her hospitalization, however suffered a fatal intracranial hemorrhage five months later.

## 3. Discussion

We present this case as a caution to our colleagues about the increased risk of noncardiac paradoxical emboli in patients with liver disease, since this abnormality may be present in up to 20% of cirrhotic patients [1, 2]. While there is ample data to support the relationship between cardiac disease and paradoxical emboli, we find nothing in the peer-reviewed literature regarding intrapulmonary shunts secondary to hepatic disease. Right-to-left vascular shunt in the adult is either interatrial or intrapulmonary, and often congenital. The acquired intrapulmonary shunt caused by cirrhosis is underappreciated but welldefined by the hepatopulmonary syndrome (HPS). A diagnosis of HPS can be established in the presence of chronic liver disease, increased alveolar-arterial gradient, intrapulmonary vascular dilatation, and absence of primary cardiac or pulmonary disease [3]. 

HPS is a secondary disorder or pulmonary vasculature characterized by intrapulmonary vascular dilatation, transpulmonary shunt, and arterial hypoxemia in the setting of chronic liver disease [4]. Despite the presence of normal parenchymal cytoarchitecture, pulmonary capillaries may dilate four- to sixfold in HPS and cause true arteriovenous communication [5]. It is believed that pulmonary microvascular dilatation is caused by abnormal vasodilator production in the setting of chronic liver disease. Hypoxemia is the most common clinical manifestation and is the result of inefficient gas exchange in dilated pulmonary vessels and intrapulmonary arteriovenous shunting. Moreover, abnormal vascular anatomy can impair the filtering function of pulmonary microcirculation and lead to direct shunting between pulmonary and systemic circulations. 

We acknowledge in this case that the evidence for microemboli resulting from pulmonary shunt is not as robust as we would have liked. Embolic signals may also be the result of intra-arterial angiography with mobilization of arterial thrombotic material. Further, the cessation of the signals on aspirin might also be compatible with arterial thrombotic emboli. However, the existence of right-to-left shunt in this case is not disputed and the remaining explanations would be self-limiting and likely not require additional management.

Our paper represents the first description of HPS in the neurologic literature with real-time evidence of transpulmonary shunting and concurrent paradoxical cerebral microemboli. We introduce this case report to compel practitioners to consider paradoxical emboli resulting from HPS in cirrhotic patients since it can often be difficult to elicit subtle neurologic changes on clinical examination of patients with end stage liver disease. Because many of these patients will have altered mental status or unable to fully participate in clinical examination, ischemic stroke may be missed. If intrapulmonary shunt can be confirmed as the source of cerebral emboli, there may be additional treatment options to traditional antiplatelet therapy which would certainly be preferable in the setting of symptomatic hepatic disease. HPS is progressive but can be reversible, and pulmonary angiography enables the treatment of arteriovenous fistula to be carried out by embolization.
